# Emerging role of radiolabeled nanoparticles as an effective diagnostic technique

**DOI:** 10.1186/2191-219X-2-39

**Published:** 2012-07-18

**Authors:** AndréLuís Branco de Barros, Andrew Tsourkas, Babak Saboury, Valbert Nascimento Cardoso, Abass Alavi

**Affiliations:** 1 Pharmacy School, Universidade Federal de Minas Gerais, Belo Horizonte, Minas Gerais, 31270-910, Brazil; 2 Department of Radiology, Hospital of the University of Pennsylvania, Philadelphia, PA, 19104, USA; 3 Department of Bioengineering, University of Pennsylvania, Philadelphia, PA, 19104, USA

**Keywords:** nanoparticles, PET imaging, SPECT imaging, tumor, inflammation, cardiovascular disease

## Abstract

Nanomedicine is emerging as a promising approach for diagnostic applications. Nanoparticles are structures in the nanometer size range, which can present different shapes, compositions, charges, surface modifications, *in vitro* and *in vivo* stabilities, and *in vivo* performances. Nanoparticles can be made of materials of diverse chemical nature, the most common being metals, metal oxides, silicates, polymers, carbon, lipids, and biomolecules. Nanoparticles exist in various morphologies, such as spheres, cylinders, platelets, and tubes. Radiolabeled nanoparticles represent a new class of agent with great potential for clinical applications. This is partly due to their long blood circulation time and plasma stability. In addition, because of the high sensitivity of imaging with radiolabeled compounds, their use has promise of achieving accurate and early diagnosis. This review article focuses on the application of radiolabeled nanoparticles in detecting diseases such as cancer and cardiovascular diseases and also presents an overview about the formulation, stability, and biological properties of the nanoparticles used for diagnostic purposes.

## Review

### Introduction

Nanomedicine can be defined as the use of nanoparticles for diagnosis, monitoring physical and pathologic processes, for therapy, and for control of biological systems [1]. Nanoparticles are defined as structures that are nanometers in size (often smaller than 100 nm) [2-4]. As small structures, they extravasate through the endothelial cell layers and interact with the cell structures of various tissues, but they are also large enough to transport high payloads of therapeutic or diagnostic agents (e.g., radioisotopes for molecular imaging) [5]. In contrast to atoms and traditional molecules, nanomaterials have an immense available surface area per unit of volume and tunable optical, electronic, magnetic, and biologic properties. They can be engineered to have different sizes, shapes, chemical compositions, surface chemical characteristics, and hollow or solid structures [6,7].

Nanoparticles can be made of materials of diverse chemical nature, the most common being metals, metal oxides, silicates, polymers, carbon, lipids and biomolecules. Nanoparticles exist in several different morphologies, such as spheres, cylinders, platelets, and tubes. Generally, they are designed with surface modifications tailored to meet the needs of specific applications [8]. When used for *in vivo* studies, nanoparticles are typically coated with a hydrophilic polymer on their surface. The most broadly used polymer is polyethylene glycol (PEG), which reduces their uptake by the reticuloendothelial system (RES) and increases circulation time when compared with uncoated counterparts [9-11]. In addition, with this approach, aggregation between particles and association with serum and tissue proteins are diminished. Furthermore, solubility in serum increases due to the hydrophilic character of ethylene glycol units. As a result, liver uptake for those particles is significantly lower than that of non-PEGylated nanoparticles, allowing nanoparticles to remain in the blood pool for an extended period of time [12].

**Figure 1:**
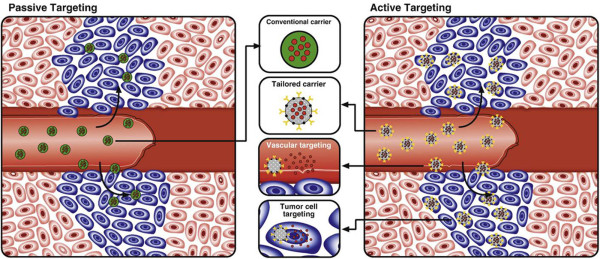
**Passive versus active targeting.** (Left) In passive targeting, particles tend to passively diffuse through the leaky vasculature of the tumor bed and accumulate primarily through the enhanced permeability effect. (Right) In active targeting, once particles have extravasated in the target tissue, the presence of ligands on the particle surface facilitates their interaction with receptors that are present on tumor or other cells, resulting in enhanced accumulation and preferential cellular uptake through receptor-mediated processes. This approach can be used either for vascular targeting and/or tumor cell targeting purposes. Reproduced with permission from [17].

Thus far, the most prominent areas where the utility of nanomedicine has been explored are for treatment and imaging of cardiovascular diseases and cancer [13]. Two approaches are generally used to access these tissue structures. First, nanoparticles can take advantage of the increased vascular permeability and pass through the fenestrations that are present in vessels. Such spontaneous accumulation or passive targeting is known as the enhanced permeability and retention effect (EPR effect). [14-16]. Second, molecules can be attached to the surface of these nanoparticles to target specific cells or tissues (active targeting) (Figure 1) [17]. A diverse range of molecules has already been used to achieve higher drug concentration in diseased tissues, including V-CAM, I-CAM, folate, peptides, antibodies, etc. [18-21]. In addition, nanoparticles have been prepared with two different ligands. This dual ligand approach has demonstrated success in improving the selectivity when compared to a single ligand approach [22,23].

**Figure 2:**
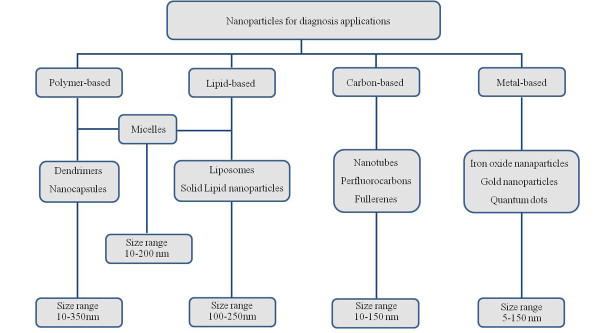
The most common nanoparticles reported for diagnostic purposes.

This review will focus on radiolabeled nanoparticles for diagnostic applications since we consider them to be a promising approach for early diagnosis (Figure 2). Radiotracer-based imaging either using single-photon emission computed tomography (SPECT) or positron-emission tomography (PET) is particularly suited for examining targeted *in vivo* molecular imaging approaches. The major advantages of SPECT and PET molecular imaging techniques over other approaches are that they are highly sensitive and specific, allow accurate quantification, and there is no limit to tissue penetration in any organ. The characteristics of each modality are summarized in Table 1[24-26]. Gamma emitters with energies in the range of 100 to 300 keV can be used for planar imaging or SPECT imaging, and a variety of positron-emitting radionuclides appears suitable for PET studies (Table 2) [27].

**Table 1:** **Characteristics of imaging techniques**[20]

**Imaging modality**	**Spatial resolution**	**Depth of penetration**	**Sensitivity (mol/L)**	**Molecular probe**
PET	1 to 2 mm	No limit	10^−11^ to 10^−12^	ng
SPECT	0.3 to 1 mm	No limit	10^−10^ to 10^−11^	ng
FMT (optical)	1 to 3 mm	<5 cm	10^−6^ to 10^−12^	μg
MRI	50 to 250 μm	No limit	10^−3^ to 10^−5^	μg to mg
X-Ray, CT	25 to 150 μm	No limit	-	mg
Ultrasound	30 to 500 μm	mm to cm	10^−6^ to 10^−9^	μg to mg

PET, positron emission tomography; SPECT, single-photon emission computed tomography; FMT, fluorescence molecular tomography; MRI, magnetic resonance imaging; CT, computed tomography.

**Table 2:** **Most common radionuclides for scintigraphic imaging**[23]

**Radionuclides**	**Emission type**	**Half-life**	***E***_**max**_**(*γ*) [keV]**
^131^I	γ (81.2%), β	8.0 days	284, 364, 637
^67^ Ga	γ	78.3 h	93, 184, 300, 393
^111^In	Auger, γ	67.2 h	171, 245
^123^I	Auger, γ	13.2 h	159
^99m^Tc	γ	6.0 h	140
^18^ F	Positron	1.83 h	511
^64^Cu	Positron	12.7 h	511
^188^Re	γ (15%), β	16.9 h	155

### Liposomes

Over the past few decades, liposomes have received widespread attention as carrier compounds for therapeutic and/or diagnostic purposes. Liposomes, first described by Bangham and co-workers, are spherical vesicles which form when phospholipids are exposed to an aqueous environment [28,29]. The lipid components of liposomes are predominantly phospholipids (e.g., phosphatidylcholine, phosphatidylethanolamine). However, other compounds can be added to liposomal preparation to provide more stability to the vesicles, such as cholesterol [30]. Because of the hydrophobic lipid bilayer surrounding an aqueous core volume, liposomes are suitable for encapsulating hydrophobic agents in the lipid shell, hydrophilic agents in the aqueous core, and amphiphilic agents distributed through the hydrophobic/hydrophilic domains. Typically, liposomes can be classified according to their size, charge, and lipid composition. In regard to size, liposomes can be divided into three categories: small unilamellar vesicles or SUV (25 to 100 nm), large unilamellar vesicles or LUV (>100 nm), and multilamellar vesicles or MLV (100 to 1,000 nm) [31]. When superficial charge is considered, liposomes can be neutral or charged. Liposomes that are negatively or positively charged have been reported to have shorter half-lives, some toxicity, and rapid blood clearance [32,33]. At least three different classes of liposomal composition are described: (1) Conventional liposomes or the first generation of liposomes are composed of natural phospholipids or lipids. Addition of cholesterol to conventional liposomes has been attempted in order to improve bilayer stability. However, these kinds of liposomes have encountered several challenges, including high instability in plasma, which results in short blood circulation half-life [9,34]. (2) Stealth liposomes or long-circulanting liposomes were synthesized by conjugating hydrophilic polymers, which increase their circulation half-life [9-11]. (3) Targeted liposomes have been engineered with different types of target moieties, such as antibodies, peptides, folic acid, and carbohydrate. Indeed, targeted liposomes are able to increase the concentration of the therapeutic or diagnostic agent in specific tissues/cells that overexpress the target receptors, antigens, or unregulated selectins [35-38]. A new generation of liposomes (e.g., stimuli-sensitive) has been developed in order to improve drug delivery. One example is a pH-sensitive liposome used to improve the endosomal release of a drug-loaded liposome into the cytoplasm [39,40].

**Figure 3:**
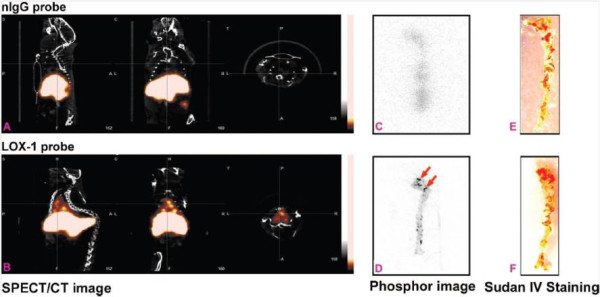
**Images of mice injected with **^**111**^**In-loaded liposomes.** (**A**) SPECT and (**C**) *ex vivo* phosphor imaging showed no focal, aortic arch hot spots in ApoE −/− mice injected with the nIgG probe, whereas all ApoE −/− mice injected with the LOX-1 probe revealed hot spots in the aortic arch ((**B**) includes sagittal, coronal, and transverse planes), confirmed by (**D**) *ex vivo* phosphor imaging. Sudan IV staining demonstrated comparable plaque distribution pattern for the (**E** and **F**) two groups. Reproduced with permission from [47].

Liposomes labeled with radioisotopes have been used for PET and SPECT imaging [29,41-45]. Dams and co-workers have demonstrated the use of ^99m^Tc-PEG liposomes for scintigraphic detection of inflammation and infection. These particles take advantage of the high permeability to extravasate to sites of inflammation and/or infection. A high correlation was observed between scintigraphic results and biopsy, culture, surgery, and follow-up to 6 months. It was found that infection and inflammatory sites could be imaged with radiolabeled liposomes [46]. Recently, Li et al. have reported the use of indium (^111^In)-loaded liposomes, surface-targeted with antibodies to the low-density lipoprotein receptor LOX-1. These liposomes were successfully used to acquire molecular images of atherosclerotic plaques in ApoE −/− mice. Mice injected with control liposomes, coated with nonspecific IgG (nIgG), did not exhibit any detectable signal in the atherosclerotic plaques (Figure 3) [47]. Examples reported in this article, containing animal or human studies, had been approved by an appropriate ethics committee.

**Figure 4:**
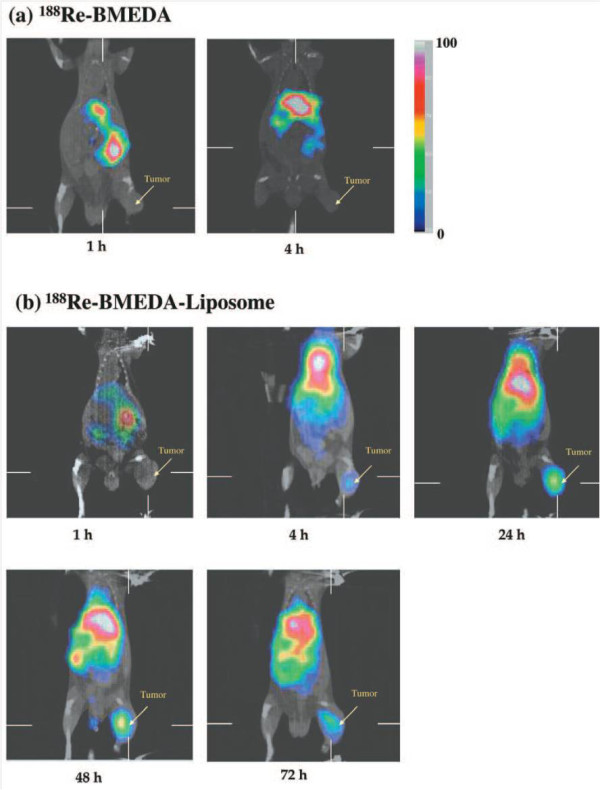
**MicroSPECT/CT images of C26 tumor-bearing BALB/c mice following injection of **^**188 **^**Re-BMEDA-liposomes or**^**188 **^**Re-BMEDA.** (**a**) Images of mice at 1 and 4 h after i.v. injection of ^188^Re-BMEDA. (**b**) Images of mice at 1, 4, 24, 48, and 72 h after injection of ^188^Re-BMEDA-liposome. Reproduced with permission from [49].

Studies conducted by Harrington et al. have demonstrated the potential application of liposomes in tumor diagnosis. Seventeen patients with different types of cancer were evaluated by scintigraphic imaging after injection of ^111^In-DTPA-labeled PEGylated liposomes. Positive images were obtained in 15 studies. This data suggested that liposomes can be an interesting vehicle for delivering diagnostic agents to tumors [48]. Another study reported the biodistribution of ^188^Re-*NN*-bis(2-mercaptoethyl)-*N′,N′*-diethylethylenediamine (BMEDA)-labeled PEGylated liposomes in murine C26-colon tumor-bearing mice. MicroSPECT/computed tomography (CT) images were acquired from 1 to 72 h. Image analysis revealed that liposomes had a higher tumor uptake at all time points when compared with unencapsulated ^188^Re-BMEDA (Figure 4). Biodistribution data were used to confirm higher uptake at 24 h post-injection (3.62 ± 0.73% injected dose (ID)/g) when compared with unencapsulated radiopharmaceutical (0.51 ± 0.06% ID/g) [49].

**Figure 5:**
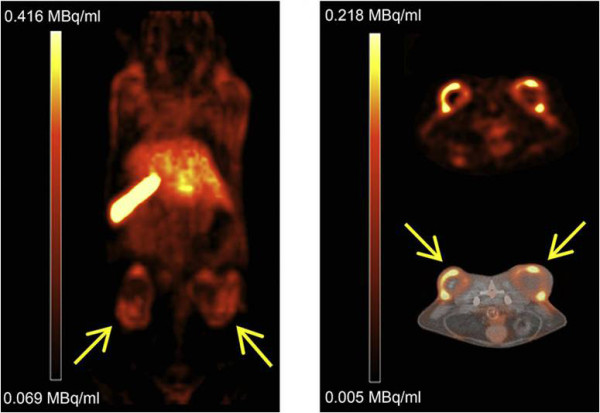
**PET/CT images of **^**64**^**Cu liposome distribution in HT29 tumor-bearing mice.** Tumors were implanted on the right and left flanks. Coronal PET image 24 h after injection (left). Axial PET image (right top) and axial PET/CT fusion (right bottom) images 24 h after injection. Adapted with permission from [50].

Typically, positron emitters have short half-lives (Table 2). As a result, in selecting the appropriate candidates, preparation schemes that are fast and least complicated should be considered as liposome tracers. In this context, Petersen et al. have reported recently a successful method to rapidly encapsulate radionuclides (remote loading method), showing a feasible way to obtain ^64^Cu-loaded PEGylated liposomes with high loading efficiency. Biodistribuition studies showed high tumor uptake at 24 hours post-liposome injection (5.0% ID/g). MicroPET/CT images visualized an implanted colon adenocarcinoma in a mouse model at 24 h post-liposome injection (Figure 5) [50].

### Iron oxide nanoparticles

Over the past two decades, iron oxide nanoparticles have received enormous attention for imaging applications. These nanoparticles can be divided into two categories based on their structural configuration: (1) a magnetic particle core (typically magnetite, Fe_3_O_4_, or maghemite, Fe_2_O_3_) coated with a hydrophilic and biocompatible polymer, such as PEG, dextran, alginate, and poly(dl-lactide-*co*-glycolide); and (2) a porous biocompatible polymer in which iron oxide nanoparticles are entrapped within the polymer matrix [51]. The coating process is important to provide colloidal stability, biocompatibility, and reduced uptake by the RES. The presence of a coating material is essential to avoid the premature clearance from the blood [52]. Generally, coated and neutral surfaces are preferred to minimize opsonization and clearance [53]. In addition to the coating material, nanoparticle size also plays a critical role in dictating blood circulation times since, in general, nanoparticles larger than 200 nm are rapidly removed from bloodstream via the RES system, and nanoparticles smaller than 10 nm undergo renal filtration. Therefore, nanoparticles with mean diameters between 10 and 100 nm are generally preferred since they can attain much longer circulation times and, consequently, can achieve higher accumulation at the target site [54,55].

In regard to their hydrodynamic diameter, iron oxide nanoparticles can be classified into superparamagnetic iron oxide (SPIO) (60 to 250 nm), ultra small paramagnetic iron oxide (USPIO) (5 to 40 nm), and micrometer-sized particles of iron oxide (MPIO) (0.9 to 8 μm). USPIO can be further chemically modified, termed cross-linked iron oxide nanoparticles (CLIO), in order to attach new molecules for targeting purposes [56-58]. Several studies have been reported using CLIO for the active targeting of various diseases, including cancer and atherosclerosis [59-64].

Because of their magnetic properties, iron nanoparticles have been studied extensively as magnetic resonance imaging (MRI) contrast agents. Nevertheless, some studies have reported their use for both MRI and SPECT or PET [65-69]. This approach poses numerous benefits since it takes advantage of high PET sensitivity and the high spatial resolution of MRI. As a result, it should theoretically be possible to obtain ‘perfect’ spatial registration of molecular/functional PET and anatomic/functional MRI [70-72].

**Figure 6:**
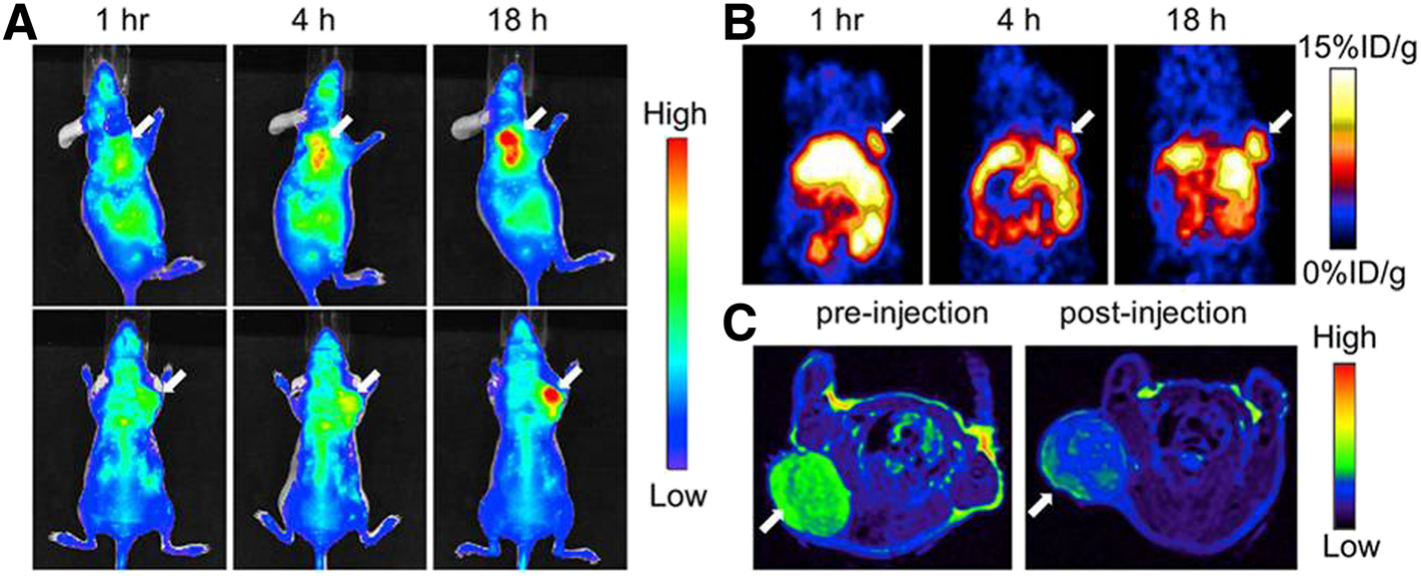
***In vivo *NIRF (a) and PET (b) images of mouse injected with iron oxide nanoparticles.** Images were acquired 1, 4, and 18 h after injection. **(c)** MRI images acquired before and 18 h after injection. Reproduced with permission from [73].

Lee et al. have reported a radiolabeled iron oxide nanoparticle conjugated with cyclic arginine-glycine-aspartic (RGD). These particles were functionalized with DOTA for labeling with ^64^Cu. The iron nanoparticles, which had a hydrodynamic diameter of 45 nm, showed high avidity for the integrin α_v_β_3_ in a competitive binding assay. In addition, PET and MR images were acquired showing delivery of RGD-iron nanoparticles to U87MG human glioblastomas in tumor-bearing mice. The highest uptake at the tumor site was achieved at 4 h post-injection (10.1% ID/g). When RGD receptors were blocked with unconjugated RGD, the tumor uptake was significantly reduced, confirming specificity for integrin α_v_β_3_[18]. Recently, Xie et al. have performed PET/near-infrared fluorescence (NIRF)/MRI tri-functional iron oxide nanoparticles for tumor detection. The particles were labeled with ^64^Cu-DOTA and Cy5.5 and tested in a subcutaneous U87MG xenographic mouse model. For PET/NIRF/MRI images, clear tumor delineation was observed by all three imaging modalities (Figure 6). Compared with NIRF results, the PET imaging showed much higher tumor-to-muscle ratios, reaching 8.28% ID/g at 18 h [73].

**Figure 7:**
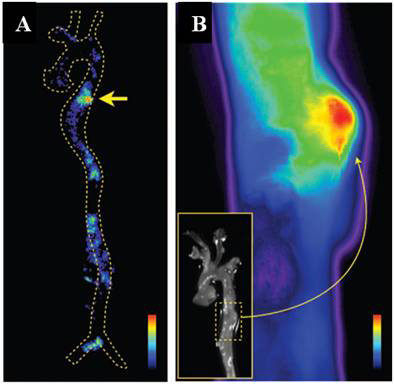
**Autoradiography and fluorescence reflectance image of the aorta.** (**A**) Autoradiography at an aneurysm in the descending thoracic aorta (arrow). (**B**) Fluorescence reflectance image of the same aorta. Nuclear and optical imaging concordantly showed nanoparticle accumulation in the aneurysmatic vessel wall. Adapted with permission from [75].

A dextran-coated and DTPA-modified magnetofluorescent 20-nm nanoparticle was prepared and radiolabeled with ^64^Cu to yield a PET, magnetic resonance, and optically detectable imaging tracer for macrophages in inflammatory atherosclerosis in ApoE −/− mice. Biodistribution studies revealed that the percent injected doses accumulated in aortas and carotid arteries were 260% and 392% higher, respectively, than in wild-type mice. PET/CT images showed robust signal in the aortic root and arch (mouse atheroma), showing a target-to-background ratio equal to 5.1 [74]. Another study recently published by Nahrendorf and co-workers used ^18^ F-CLIO for detection of macrophages in aortic aneurysms induced in ApoE −/− mice. The nanoparticles were also labeled with a near-infrared fluorochrome. PET/CT images showed significantly higher uptake in the aneurismal aortic section compared with what was found in the wild-type aorta. In addition, the PET signal within the aneurysm was stronger than in atherosclerotic plaques. *Ex-vivo* imaging by autoradiography and fluorescence reflective microscope confirmed high uptake of ^18^ F-CLIO in the aneurysm (Figure 7) [75].

### Gold nanoparticles

Gold nanoparticles have received special attention in the biomedical field due to their biocompatibility, facile conjugation to biomolecules, and the unique optical properties conferred by their localized surface plasmon resonance [76]. Moreover, gold is resistant to oxidation under physiological or ambient conditions, which permit unrestricted interaction of gold with the biological environment [23]. The optical properties of gold depend on the nanoparticle size and shape. One can manipulate the shape of gold nanostructures to control their electronic and associated optical properties for the desired applications.

Gold nanoparticles can be manufactured into different shapes, such as gold nanospheres, nanorods, nanocages, and nanostars [77]. Their widespread utilization in biological assays relies on the availability of synthetic methods yielding nanoparticles with desired characteristics including high solubility in water, adequate morphology, size dispersion, and surface functionalities. The most common method to prepare gold nanoparticles is the citrate reduction method of [Turkevich 78]. This approach is very convenient since appropriate functionality can be easily applied by replacement of the citrate. The nanoparticle surface can be modified in order to achieve specific labeling with biomolecules, such as antibodies, cardohydrates, and proteins.

**Figure 8:**
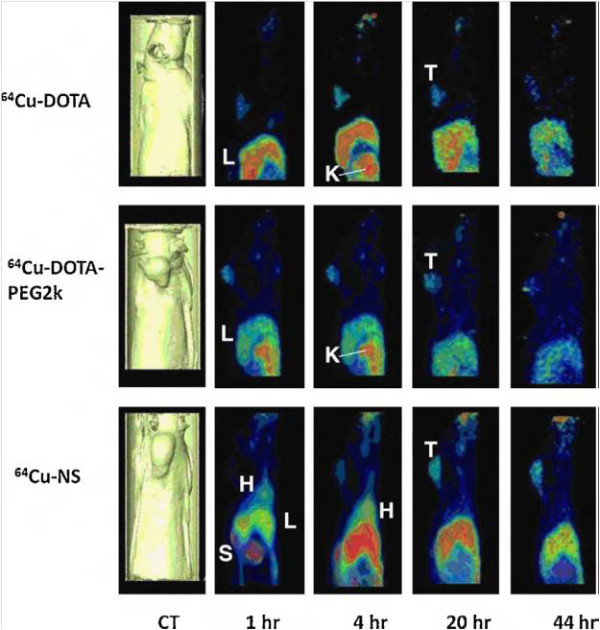
**Sagittal PET images of three rats.** The images were acquired at 1, 4, 20, 44 h after injection of ^64^Cu-DOTA, ^64^Cu-DOTA-PEG2k, and radiolabeled gold nanoshell (^64^Cu-NS), respectively. Surface-rendered CT images depicting tumor location are also shown (H, heart; L, liver; K, kidney; T, tumor). Reproduced with permission from [83].

In the imaging field, gold nanoparticles have shown promise for their use in computed tomography, Raman spectroscopy, and photoacoustic imaging. Recently, several reports have also indicated the use of gold nanoparticles labeled with gamma emitters or positron emitters for multimodality imaging, such as PET/CT imaging [79-82]. This approach takes advantage of higher PET sensitivity and an accurate localization provided by CT. In this context, Xie et al. have prepared a radiolabeled gold nanoshell for tumor diagnosis. Gold nanoparticles were coated with PEG2k-DOTA for ^64^Cu chelation. After surface modification, the nanoparticles' diameter was 170 nm, and the surface charge was −5 mV. The radiolabeling efficiency was 81.3%, and ^64^Cu binding was shown to be stable for 3 h. PET imagines were acquired at different times, using ^64^Cu-DOTA and ^64^Cu-DOTA-PEG2k as controls. The ^64^Cu-nanoparticles showed higher accumulation in the tumor site, mainly at 20 and 44 h post-injection (Figure 8) [83].

**Figure 9:**
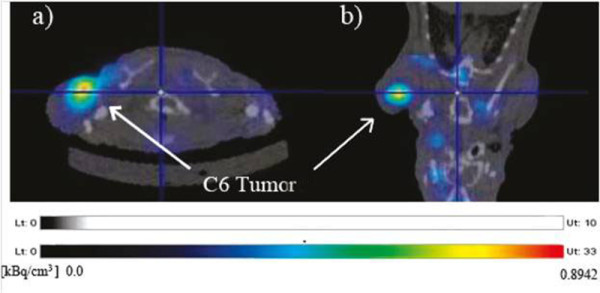
**Transaxial (a) and coronal MicroSPECT/CT (b) images of an athymic mouse with a C6-induced tumor.** The images were taken 1 h after ^99m^Tc-labeled gold nanoparticles conjugated with c[RGDfk(C)] intravenous administration. Adapted with permission from [84].

Morales-Ávila and co-workers have recently reported the use of ^99m^Tc-labeled gold nanoparticles conjugated with c[RGDfk(C)] for tumor imaging. *In vivo* studies were performed in C6 human glioma-bearing athymic mice. Nanoparticles showed rapid blood clearance, with less than 0.5% ID/g remaining in the blood compartment 1 h post-injection. However, evident tumor uptake was observed at the same time (1 h post-injection) (3.65% ID/g) (Figure 9) [84].

Shao et al. have reported ^125^I-labeled gold nanorods for imaging inflammation in an adjuvant-induced arthritic rat model. These nanoparticles were PEGylated in order to achieve long circulation and also conjugated with anti-intercellular adhesion molecule 1 antibody, which is overexpressed in arthritic diseases. Results showed higher accumulation in the inflamed joints when compared with the control group [85]. These findings further support the use of gold nanoparticles in detecting inflammation due to the increased permeability of inflamed areas, allowing for higher uptake of the diagnostic agent.

### Micelles

Micelles are self-assembled nanostructures with a hydrophobic core and hydrophilic shell which are spontaneously formed above a certain concentration (critical micellar concentration). They can be constituted from lipid-based or polymer-based amphiphilic molecules [13]. Micellar formulations, including polymer micelles, have been extensively studied as molecular delivery systems [86]. In general, the hydrophobic portion of the polymer forms a semisolid core, while the hydrophilic portion of polymer forms the corona. The hydrophobic core promotes stability, while the hydrophilic shell increases solubility, prevents opsonization, and reduces RES uptake. Molecules can be physically trapped into the hydrophobic core or can be covalently bound with components of the corona [87].

The *in vivo* performance of micelles is controlled by several factors, such as size distribution, shape, density, deformability, and surface properties which can determine physicochemical and pathophysiological interactions. Blood stability can be affected by several factors including phagocytotic/endocytotic recognition, immune responsiveness, and vascular escape routes. In addition, if these nanoparticles are charged, they will be rapidly cleared from circulation. In contrast, neutral particles display longer circulation times. In general, micelles in the range of 10 to 200 nm are preferred since they can avoid the RES more effectively, increasing circulation half-life [88-90]. In summary, the characteristics of micelles that are favorable for *in vivo* imaging include facile encapsulation of a diagnostic agent, thermodynamic stability (often a low critical micellar concentration is required), and long blood circulation times, avoiding RES recognition.

**Figure 10:**
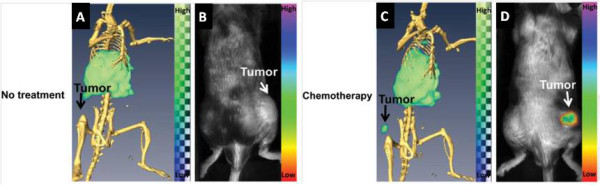
**Imaging after administration of **^**111 **^**In-labeled annexin A5-CPM in untreated and treated mice.** (**A**) Dual SPECT/CT and (**B**) near-infrared fluorescence optical imaging after administration of ^111^In-labeled annexin A5-CPM into EL4 lymphoma-bearing mice (untreated animals). (**C**) Dual SPECT/CT and (**D**) near-infrared fluorescence optical imaging of EL4 lymphoma apoptosis after injection of ^111^In-labeled annexin A5-CPM into treated mice. Adapted with permission from [95].

In this sense, some radiolabeled micelles have been prepared for diagnostic purposes [91-94]. Zhang et al. have prepared peptide-conjugated polymeric micellar nanoparticles which were used to identify EphB4 receptors in prostate cancer in a mouse model by SPECT [93]. The same group has recently reported the use of annexin A5-conjugated polymeric micelles (CPM) for the detection of apoptosis. Annexin A5 is a protein which binds strongly and specifically to phosphatidylserine residues. These residues are present on the cell surface in the early stage of apoptosis. Micelles were labeled with ^111^In and a near-infrared fluorescent indocyanine (Cy7)-like dye in order to acquire SPECT and optical images. Tumor apoptosis was clearly visualized in mice bearing EL4 lymphomas treated with cyclophosphamide and etoposide. On the other hand, untreated animals showed lower accumulation of the nanoparticles (Figure 10) [95]. In another study, Xiao et al. have reported a multifunctional micelle made up with a hyperbranched amphiphilic block copolymer. These particles were conjugated with cRGD peptide (for integrin α_v_β_3_ target), NOTA (a macrocyclic chelator for ^64^Cu-labeling and PET imaging), and doxorubicin (DOX) for cancer therapy. When injected into U87MG tumor-bearing mice, these particles (^64^Cu-micelle-DOX-cRGD) showed higher tumor accumulation than the nontargeted particle (^64^Cu-micelle-DOX) used as control. Furthermore, injecting a blocking dose of cRGD peptide along with ^64^Cu-micelle-DOX-cRGD reduced tumor uptake significantly, indicating integrin α_v_β_3_ specificity. These findings suggested that such multifunctional micelle approach can be considered a promising cancer theranostic platform [96].

### Carbon-based nanoparticles

The most prominent types of carbon-based nanoparticles that have been evaluated for medical applications are carbon nanotubes, fullerenes, perfluorocarbon nanoemulsions, and graphene oxide nanoparticles. Carbon nanotubes (CNTs), first described by [Iijima 97], are well-ordered hollow nanomaterials with lengths from several hundred nanometers to several micrometers and diameters of 0.4 to 2 nm for single-walled carbon nanotubes (SWNTs) and 2 to 100 nm for multi-walled carbon nanotubes (MWNTs). In recent years, efforts have been devoted to explore the potential biological applications of CNTs, which were mainly motivated by their interesting size, shape, and structure [98,99]. Some studies have reported the use of radiolabeled CNTs [100-104]. McDevitt et al. have prepared antibody-functionalized SWNTs labeled with indium-111 for tumor targeting. *In vitro* and *in vivo* studies were performed, and results suggested that CNTs could be used as a novel delivery platform [105]. More recently, Liu and collaborators have published *in vivo* biodistribution data for PEG-coated ^64^Cu-labeled SWNTs that were functionalized with RGD peptide in U87MG tumor-bearing mice. SWNT-PEG5400-RGD showed higher tumor uptake (approximately 15% ID/g at 24 h post-injection) when compared with PEG-coated SWNT free of RGD (approximately 4% ID/g at 24 h post-injection) [106]. Ruggiero et al. have reported a radiolabeled carbon nanotube that targets the tumor neovasculature via the E4G10 antibody. The E4G10 antibody specifically targets the monomeric vascular endothelial-cadherin epitope expressed on tumor angiogenic vessels. PET ROI data revealed a tumor-to-muscle ratio at 96 h post-nanotube injection equal to 5.08. This result can support the idea that CNTs can be used for medical applications, including diagnostic approaches [107].

Fullerenes were discovered in 1985 and were first studied for diagnostic purposes soon afterwards. The most representative fullerene is C_60_, which has a diameter around 0.7 nm. Fullerenes naturally have poor water solubility, resulting in the formation of aggregates when administrated in biological systems. Fortunately, this inconvenience can be solved by preparing functionalized fullerenes (e.g., -OH, -NH2, -COOH), which show adequate solubility in polar solvents [108,109]. Fullerenes can be used to entrap metals inside their cavity, leading to nanostructures called endohedral metallofullerenes [110]. One of the most important applications of this approach is in molecular imaging with radiotracers due to the fact that it is possible to encapsulate radioactive metals for diagnostic purposes [111-113]. Nickolic et al. have described the preparation and biodistribution of ^125^I-radiolabeled fullerenes. The results confirmed the possibility of using these molecules as radiotracers for *in vivo* studies [114].

Perfluorocarbon nanoemulsions are a multifunctional technology that are typically about 250 nm in diameter and have a perfluorocarbon core wrapped by a monolayer of phospholipid. Different perfluorocarbons can be used for the core, including perfluorodichlorooctane, perfluorodecaline, perfluoro 15-crown-5 ether (CE), and, most commonly, perfluorooctyl bromide (PFOB). The biocompatibility of PFOB is well documented showing no toxicity, even at large doses. The large surface area of nanoparticles can easily accommodate 100 to 500 radionuclides, allowing their application for diagnostic purposes [115,116]. Hu et al. have developed a α_v_β_3_-integrin-targeted ^111^In-perfluorocarbon nanoparticle for detecting tumor angiogenesis in rabbits. The tumor uptake in rabbits receiving α_v_β_3_-integrin-targeted nanoparticles was four-fold higher than that in the nontarget control at 18 h [117].

**Figure 11:**
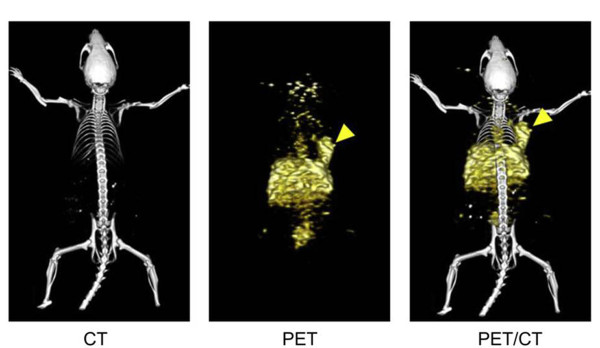
**Representative PET/CT images of **^**66 **^  **Ga-labeled nanographenes in 4T1 tumor-bearing mice at 3 h after injection.** Tumor site is indicated by arrowheads. Adapted with permission from [126].

Graphene oxide is a class of dual-dimensional carbon-based nanoparticles which has attracted attention due to its unique electronic, thermal, mechanical, and optical properties [118]. Several studies have reported a variety of graphene-based platforms useful for biomedical applications, including bioimaging [119-123]. The toxicity of graphene is closely related to its surface characteristics. As a result, PEG functionalized graphene oxide nanoparticles have showed minimal toxicity when administrated in mice [124]. Zhang and co-workers have reported graphene oxide nanoparticles which exhibited long blood circulation and low uptake by MPS, indicating that those particles might be used for biomedical applications [125]. Hong et al. have prepared ^66^ Ga-labeled nanographene for tumor vasculature imaging. These particles were conjugated with an antibody that binds to CD105 (a marker for tumor angiogenesis) and then injected into 4T1 tumor-bearing mice. PET-CT images revealed tumor uptake, indicating nanographene affinity to tumor vasculature (Figure 11) [126].

### Other nanoparticles

Besides the various nanoparticles presented in the previous sections, in order to look into new possibilities in nanotechnology-based diagnosis, other nanoparticles have been reported including quantum dots, dendrimers, nanocapsules, solid lipid nanoparticles, and silica nanoparticles.

Quantum dots are semiconductor nanocrystals made from a variety of different compounds, such as cadmium and selenide with unique optical and electrical properties [1,13,127]. Most quantum dots are composed of heavy metals that are not normally exocytosed from cells, and this can give rise to *in vivo* cytotoxicity and limited clinical safety. Nevertheless, interest in these structures has resurged with the advent of heavy metal-free quantum dots, which are expected to have less toxicity than earlier designs [128]. A few radiolabeled quantum dots have been reported with a variety of isotopes, such as ^125m^Te, ^18^ F, and ^64^Cu [129-132]. Cai et al. have reported an amine-fuctionalized quantum dot modified with RGD peptides and DOTA chelator for PET/NIRF imaging of the integrin α_v_β_3_. Results showed high linear correlation between nuclear and optical signals. In this study, tumor uptake was 4% ID/g at 18 h post-injection [133]. Similarly, Chen et al. have prepared quantum dots conjugated with vascular endothelial growth factor (VEGF) for imaging angiogenic vessels within tumors. Both NIR fluorescence imaging and PET imaging showed specific delivery of nanoparticles to sites of VEGF receptor over expression [134].

Dendrimers are a class of well-defined nanostructured macromolecules that possess a treelike architecture distinguished by exponential numbers of discrete dendritic branches radiating out from a core [135]. Dendrimers varying in their initiator core, repeating units, terminal functionality, charge, and solubility profile have been synthesized thus far [136,137]; have found diversified applications ranging from the paint industry to desalination plants; and have been tested as diagnostic and drug delivery agents [138]. These nanostructures provide three regions for modification with bioactive agents, namely, the core, branching zone, and branch surface [13]. A few radiolabeled dendrimers have been reported and found to be useful for SPECT and PET imaging studies [139-143]. Almutairi et al. have published the efficacy of biodegradable dendritic structures surface-modified with a cyclic RGD peptide and with encapsulated ^76^Br for the targeted delivery and PET imaging of hindlimb ischemia in mice [144]. Zhang et al. have reported the synthesis, biodistribution, and SPECT imaging of three radiolabeled dendrimer conjugates (^99m^Tc-dendrimer, ^99m^Tc-dendrimer-folic acid, ^99m^Tc-dendrimer-PEG-folic acid) in KB tumor-bearing mice. The ^99m^Tc-labeled PEGylated dendrimer PAMAM-folic acid conjugates showed higher tumor uptake at 6 h post-injection (10.27% ID/g) when compared with ^99m^Tc-dendrimer-folic acid (6.78% ID/g) and ^99m^Tc-dendrimer (4.38% ID/g). The MicroSPECT imaging corroborated well with biodistribution data [145].

Nanocapsules are nanoparticules composed of a core, mainly lipophilic, surrounded by a polymeric wall with lipophilic and/or hydrophilic surfactants at the interface [146]. Some studies have been reported using radiolabeled nanocapsules for *in vitro* and *in vivo* evaluations [147-150]. Pereira et al. have developed a PEG surface-modified nanocapsule labeled with technetium-99 m for detecting sites of inflammation. Results showed higher accumulation in inflamed foci than in control tissue, suggesting that such nanostructures can be useful for diagnosing inflammation [151].

Solid lipid nanoparticles are nanoscaled lipid matrices, solid at physiological temperatures and stabilized by surfactants. These nanoparticles are composed of physiological lipids (such as fatty acids and phospholipids) and tend to show high compatibility and biodegradability [152]. Recently, Andreozzi and co-workers have published a novel method to radiolabel solid lipid nanoparticles with ^64^Cu. The mean diameter of the nanoparticles was approximately 150 nm by dynamic light scattering, and *in vivo* studies showed a blood half-life of 1.4 h [153]. Andreozzi's results suggested that solid lipid nanoparticles can be potentially used for diagnostic purposes, suggesting a new field for solid lipid nanoparticles.

**Figure 12:**
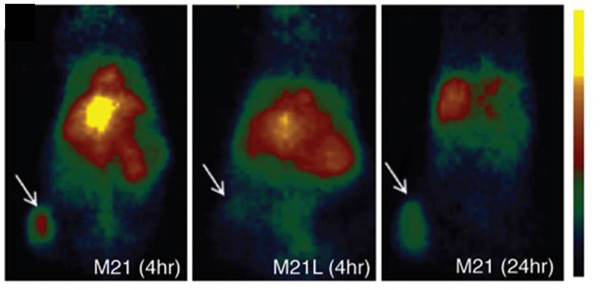
**Representative whole-body coronal microPET images.** The images were taken 4 h after injection, demonstrating M21 (left, arrow), M21L (middle, arrow), and enhanced M21 tumor contrast at 24 h after injection (right, arrow). Adapted with permission from [159].

Silica nanoparticles have gained extensive attention in biomedical field since they can be utilized in both diagnostic and therapeutic domains [154]. In addition, recent improvements in regulating the geometry, porosity, and surface characteristics of these particles have further enhanced and facilitated their biomedical applications [155-158]. Benezra et al. have reported multimodal silica nanoparticles (c-dots) conjugated with cRGD peptide and labeled with Cy5 dye and ^124^I for fluorescence and PET imaging, respectively. These 7-nm particles exhibited high-affinity/avidity binding to cells lines, which overexpress α_v_β_3_ integrin receptors (M21, HUVEC). Furthermore, when injected into M21 tumor-bearing mice, those particles were able to identify the tumor site, yielding a tumor uptake of 3.6% ID/g (Figure 12). In contrast, after injection into mice with M21L tumor (a α_v_β_3_ integrin nonexpressing cell line), tumor uptake was reduced significantly to 0.7% ID/g, indicating specificity to α_v_β_3_ integrin receptors. The US Food and Drug Administration have approved the first human clinical trial of c-dots. The planned clinical trial will involve patients with melanoma, is expected to verify that the dots are both safe and effective in humans, and also will provide promising data for potential future applications [159].

## Conclusions

Nanomedicine has received increasing interest during the past decade since it has become clear that nanoparticles can be used to circumvent some of the difficulties that are associated with the administration of standard drugs. One of the greatest advantages of this field is the ability to generate particles functionalized with a wide variety of targeting ligands and physicochemical properties. This versatility permits the creation of agents that are specifically tailored for each application. In the diagnostic field, radiolabeled nanoparticles have recently emerged as potentially very promising agents for an accurate, reliable, and early diagnosis for several disorders and diseases. These particles provide a means for performing multimodality imaging, which will further enhance the rapidly evolving synergy among different techniques (such as, PET/SPECT, MRI, CT, and NIRF). To date, the two major areas where nanoparticles have been applied are cancer and cardiovascular diseases, and some promising results have been reached in both fields. However, in order to attain a nanocarrier with suitable characteristics for biological applications, several factors must be considered. First, nanoparticle characterization should be extensively evaluated before performing animal experiments since *in vivo* performance is strongly related to shape, charge, surface modification, and size. Second, *in vivo* stability should be determined since some nanoparticles can be disrupted in the bloodstream. For instance, micelles can decompose back to their unimer forms due to the high plasma dilution. Third, it is extremely important that the radioisotope chelation with diagnostic agents remains stable over the course of imaging. Otherwise, biodistribution and imaging data will not be useful for predicting the fate of nanoparticles, due to the fact that the radioisotope distribution will no longer reflect that of the nanoparticle. In this sense, an appropriate design and use of chelators with high affinity for each isotope should be considered in order to achieve high stability and dependable data. Therefore, strong efforts should be made in order to prepare nanoparticle-based agents which can allow for efficient, specific *in vivo* delivery of diagnostic agents without systemic toxicity. Although some particles are in clinical trials, such as c-dots, most radiolabeled nanoparticles reported up to now face the challenges and are still in a preclinical stage. We believe that the future of nanomedicine for diagnostic applications lies on the use of multimodal approach by combining different imaging modalities to achieve an accurate diagnosis in cancer, cardiovascular disease, and other disorders. However, although clearly much remains to be done before radiolabeled nanoparticles can be widely adopted for routine clinical applications due to their unusual characteristics, including their extraordinary sensitivity in visualizing the targeted tissues compared to other diagnostic agents, it is likely that such preparations will have a major impact on the diagnostic arena in the near future.

## Competing interests

All authors declare that they have no competing interests.

## Authors' contributions

ALBB reviewed the current literature and wrote the manuscript. BS, VNC, and AT critically reviewed the manuscript. AA provided the concept for the review and critically reviewed and edited the manuscript. All the authors read and approved the final manuscript.
